# Mapping service activity: the example of childhood obesity schemes in England

**DOI:** 10.1186/1471-2458-10-310

**Published:** 2010-06-04

**Authors:** Catherine Aicken, Helen Roberts, Lisa Arai

**Affiliations:** 1 Centre for Sexual Health and HIV Research, Research Department of Infection and Population Health, University College London, Mortimer Market Centre, off Capper Street, London WC1E 6JB, UK; 2 General and Adolescent and Paediatrics Unit, University College London Institute of Child Health, 30 Guilford Street, London WC1N 1EH, UK; 3 School of Health and Social Care, Teesside University, Middlesbrough, Tees Valley, TS1 3BA, UK

## Abstract

**Background:**

Childhood obesity is high on the policy agenda of wealthier nations, and many interventions have been developed to address it. This work describes an overview of schemes for obese and overweight children and young people in England, and the 'mapping' approach we used.

**Methods:**

Our search strategy, inclusion criteria and coding frame had to be suitable for describing a potentially large number of schemes within a short timeframe. Data were collected from key informants, scheme publicity and reports, and via a web-survey. To be included, schemes had to be based in England, follow a structured programme lasting at least two weeks, promote healthy weight, and be delivered exclusively to overweight and/or obese children and young people (age range 4-18). Data were entered into a coding frame recording similar information for each scheme, including any underpinning research evidence, evaluation or monitoring reports. Priority questions were identified in consultation with colleagues from the Department of Health and the Cross Government Obesity Unit.

**Results:**

Fifty-one schemes were identified. Some operated in multiple areas, and by using estimates of the number of schemes provided by multi-site scheme leads, we found that between 314 and 375 local programmes were running at any time. Uncertainty is largely due to the largest scheme provider undergoing rapid expansion at the time of the mapping exercise and therefore able to provide only an estimate of the number of programmes running.

Many schemes were similar in their approach, had been recently established and were following NICE guidelines on interventions to promote healthy weight. Rigorous evaluation was rare.

**Conclusions:**

Our methods enabled us to produce a rapid overview of service activity across a wide geographic area and a range of organisations and sectors. In order to develop the evidence base for childhood obesity interventions, rigorous evaluation of these schemes is required. This overview can serve as a starting point for evaluations of interventions to address obesity. More generally, a rapid and systematic approach of this type is transferable to other types of service activity in health and social care, and may be a tool to inform public health planning.

## Background

In the UK, there is considerable policy and research interest in childhood obesity. Identified as a policy priority in 2004[1], NICE guidelines relevant to childhood and adult obesity were produced in 2006[2], and the Cross-Government Obesity Unit was established in 2008[3]. Several reviews of evidence on overweight and obesity have been published[2,4-8], addressing lifestyle and behavioural interventions for prevention and treatment. The government's Foresight report on obesity, based on a series of evidence reviews, took a broader view, specifically exploring the 'obesogenic' environment and international comparisons of trends and determinants[9].

Obesity and overweight contribute to, and result from, health inequalities. The recent House of Commons Health Committee's report on Health Inequalities[10], while commenting favourably on policy commitments to reduce health inequalities, was critical of the ways in which policy and associated practice interventions have been implemented, many without rigorous evaluation. The committee referred to the tendency for interventions to be implemented and evaluated without the collection of baseline data, with sample sizes too small to demonstrate effects, for evaluations to comprise only process evaluation or measure only 'soft' indicators, and for lacking a clear definition of objectives at the outset. This article considers some of these issues.

At a regional or national level, knowledge of existing service provision and its evidence base is essential for effective public health planning. Guidance commissioned by the Department of Health[11], on needs assessments in sexual health, for instance, makes clear that service mapping is integral to the needs assessment process. An overview of services describes of the range of provision: e.g. components of interventions, target population(s), provider organisations, funding sources and so on. Such an overview may also include (cost-) effectiveness evidence[12] and note where evidence is weak or absent. However, despite recognition of the importance of this 'background' knowledge, there is no framework to date within which to conduct service mapping exercises systematically.

As is the case in other areas, including teenage pregnancy and HIV/AIDS, once childhood obesity was identified as a public health problem and targeted for intervention, a great deal of service activity was quickly initiated. Policy and funding drivers led to the growth of a large number of schemes addressing childhood obesity. Despite this rapid growth in interventions, comprehensive information on service activity across England had not been compiled nationally before our project was commissioned by the Department of Health. Given this, our mapping exercise, conducted in 2008, aimed to produce a broad overview of English schemes for overweight and obese children and young people.

We appreciate that 'mapping' has a range of meanings. Here, we define a mapping exercise as a means of compiling and structuring information about services or interventions. The result serves the dual purpose of presenting an overview of information in a condensed format, and providing a basis for evaluation and comparison. The methods we describe allow information about interventions or services to be gathered rapidly, and presented in a way which is potentially useful to those planning services at a regional or national level.

## Methods

Our mapping approach draws on the scoping process often used to inform systematic reviews[13], and builds on work in other areas of public health[12,14-16]. The coding frame (additional file 1 table S1) describes the data we aimed to collect, using the range of sources described below. Each scheme was treated as one item (though we often drew on several sources of information to describe it)[17].

### Phase 1: Planning stage

A search strategy, inclusion/exclusion criteria, and a coding frame were developed.

We included schemes that were:

a) based in England;

b) lasting at least two weeks;

c) structured (following a defined programme);

d) delivered to overweight and/or obese children/young people;

e) delivered to children/young people within the 4-18 age group;

f) aimed at the attainment of a healthier weight.

We excluded interventions designed to prevent overweight/obesity among children of a healthy weight. This reflects the trade-off within mapping and scoping exercises[13] between breadth (number of interventions included) and depth (information about each intervention).

In designing our search strategy, we sought to use methods which would facilitate the rapid identification of schemes for possible inclusion, and minimise bias.

The coding frame was developed to list and structure information about each project. In order to manage details of an unknown number schemes, transparent measures were needed to enable the work, which had a tight timescale, to be 'contained,' if necessary, through prioritization. In consultation with policy colleagues, we gave priority to questions about the scheme's location, lead organisation, target group and evidence base.

Our inclusion criteria and coding frame have parallels with other frameworks for describing intervention characteristics, such as ASTOR (aim, setting, target, objectives, resources), originally developed to describe and map HIV health promotion activity[12], and PICO(C) (population, intervention, comparison, outcomes, context) used in systematic reviews[18].

To ensure timely completion of the project, we set a date ten weeks after the start of data collection, beyond which no further schemes, or details about schemes, were entered into the database.

### Phase 2: Data collection, storage and coding

**Figure 1:**
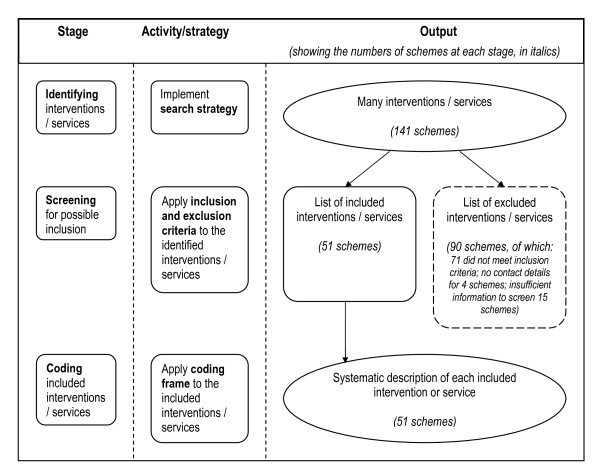
**Stages involved in data collection for a mapping exercise, showing outputs at each stage**.

Stages of data collection follow the pattern shown in Figure 1:

1. **Identifying **interventions for possible inclusion.

2. **Screening **interventions on the basis of the inclusion criteria.

3. **Coding **details about included interventions and displaying these in a structured format.

We contacted the Obesity Leads for all ten English Regions, and used an online questionnaire to collect names and contact details for schemes, and project-related reports. We contacted relevant JISCmail lists including practitioners and researchers, the Association for the Study of Obesity email list, and requested information via the Evidence Network newsletter. A web-search using mainstream and academic search engines was undertaken intending to identify schemes via their publicity, grey literature, and evaluation and research literature, and we also included schemes referred to in the Foresight report ([9] p69-70). Documents sent to us, or elicited in the course of our searches, were hand-searched for mention of other schemes, and we employed a snowball approach through which the contacts we made were asked if they knew of other schemes. With this approach we hoped to reach practitioner audiences as well as the public health, education and academic communities.

A simple internet search using the terms 'overweight' and 'obesity' was not practical, due to the huge volume of websites offering diet and weight loss advice to adults. Adding 'children' as a search term did not help given the targeting of women who have had children by the advertisers of weight loss schemes. As the volume of hits was unmanageable, we abandoned the web-search, though we acknowledge its potential usefulness in other areas of service provision.

To store details of studies, we used EPPI-Reviewer, a web application that enables researchers to manage the lifecycle of a systematic review[19], including screening and coding. Coding focussed on the priority questions that had been identified *a priori*, but where information was provided that addressed other questions, this too was included. We treated multi-site schemes with a common programme as a single entry in the database. The database constructed[20] enables users to conduct their own analyses.

### Phase 3: Analysis

We carried out a largely descriptive analysis, focusing on priority questions jointly identified with policy and research commissioners from the Department of Health.

### Ethical approval

Ethical approval for the study was obtained from the Institute of Education, University of London, where the authors were employed at the time of the study.

## Results

We identified 141 schemes for potential inclusion. Table 1 provides a breakdown of these schemes by the method by which we first found out about them. The large number of schemes identified via 'personal and telephone contacts' (83) is due to the fact that we asked all of those we contacted whether they knew about any other interventions. Via this route, we were provided with information from six local mapping exercises. These included less detail on each scheme than we required, were narrower in geographic scope, and covered a broader range of service activity (including for example health promotion activities for all ages and weights, staff training initiatives, interventions for adults). Because of this, further information was sought in order to screen and code schemes listed.

**Table 1:** Methods of identifying schemes for possible inclusion

Method by which the scheme was first identified	Number of schemes
List of schemes already known to DH	15

Web-based requests for information	20

Personal email and telephone contacts, including emails to regional Obesity Leads	83

Grey literature	3

Internet search	20

Total	141

Of these 141 schemes, 51 met our inclusion criteria, 71 were excluded, we were unable to obtain sufficient and timely information to make an informed decision on whether to include or exclude a further 15, and no contact details were found for 4 schemes. We excluded these 19 schemes (the latter two groups) from the database. Details of the included schemes are provided in additional file 2 table S2.

Some of the 51 included schemes operated at multiple sites. Through contacting providers of multi-site schemes we were able to estimate that 314 to 375 local programmes were ongoing in England. The majority of this variation is attributable to the fact that no exact figures were available from the largest scheme provider, as it was in the process of expanding in 2008, with many new local programmes being established.

Due to the predominance of this scheme provider, and the uncertainty noted, the remainder of the results use the number of scheme or intervention 'models' as a denominator (51). A typical scheme lasts the duration of a school-term, and includes components in line with guidance[2] specifying that physical activity, healthy eating and behaviour change techniques should be a part of an effective programme. All of the schemes for which we were able to obtain details on components (47 of 51) address physical activity and diet. While many schemes promote physical activity and healthy eating through educational means (45 and 46 respectively), some take a more hands-on approach to health promotion, providing opportunities for exercise; and provision, preparation or tasting of healthy foods (43; 19 respectively). We found that 37 schemes explicitly used behaviour change techniques. However this may be an underestimate since scheme publicity may have been more likely to inform children and parents about the components of the scheme, rather than the methods used. For 26 of the 51 schemes, we were able to find out the kind of health worker who delivered these components. In 21 cases, a sports/exercise worker was involved, and in 20 cases a dietician/nutritionist. After these roles, the most common were: community worker (7), school nurse (6), other health professionals (6), psychologists (3), counsellors (3) (other roles were only represented in one or two schemes). Most schemes were open to the 8-12 year age range. We obtained details of the key partner organisations for 43 schemes: most commonly these were PCTs (36 schemes) and Local Authorities (Councils - 22 schemes) - while 20 involved both of these types of organisations as key partners.

We obtained evaluation or monitoring reports for half of the schemes. This low response is attributable in part to providers considering data to be commercially sensitive; to data protection issues, particularly where there had been small numbers of participants; and to an understandable desire of those who had carried out evaluations to publish their findings themselves before making them widely available. Where a scheme's objectives were stated in reports or publicity, we recorded these, but more than half (59%, 30 schemes) did not make their objectives clear (beyond a statement, for example, that they were weight management programmes). Half of the schemes provided us with information on the outcomes measured, and this was most commonly a change in BMI percentile. It was often difficult to ascertain what evidence or information had been used in programme design, and very few had been comprehensively evaluated, which may reflect, in part, the newness of many of the schemes.

## Discussion

Providing a census of ongoing activity in a rapidly changing field is a challenge. Results are unlikely to be exhaustive or completely up-to-date due to trade-offs between coverage, the amount of detail that can be obtained, and for information to become dated as schemes begin, end and change. However, a mapping exercise of this kind provides a 'snapshot' of the range of service activity, as well as the types of services and the populations they are aimed at, and these findings can be used as a resource for public health planners, service providers and the public. Our database of schemes[20] can be used by planners and practitioners wishing to conduct searches, or their own analyses. It can also serve as a starting point for government plans to track local availability of weight management schemes[21].

We were encouraged by the enthusiastic response of many of the people with whom we had contact. However, any search strategy may introduce bias. Despite an increase in inter-sectoral working, the use of key informants predominantly from the health sector may mean that schemes implemented by teachers, youth workers or leisure centre staff were less likely to be brought to our attention. Yet schemes which do not require referral from a health professional may be more likely to be widely publicised. A bias towards well-known schemes may have been off-set by the willingness of our respondents to post our email survey link onto various 'closed' lists of practitioners. We also acknowledge a potential bias towards evaluated schemes, which tend to be better known in the field and/or have a presence in the academic literature.

Despite this possible bias, we identified evaluation as an area of concern for the majority of intervention models. What is described as 'evaluation' in many scheme reports might better be described as monitoring and the presentation of process data. Evaluations of effectiveness were often weak or, more usually, absent. Changes to interventions included activities, duration, and target group (age range and/or degree of overweight). These changes were made in response to local needs, funding pressures, and concerns about drop-out - concerns which are understandable, but which can make evaluation problematic. As we have noted, inadequate evaluation of health and social interventions can hamper attempts to address health issues and health inequalities. Problems with drop-out were mentioned to us several times, and it would have been extremely challenging to collate a measure of the overall numbers and demographics of scheme participants, as this would be reliant on the level of attendance considered 'adequate' among schemes which varied in duration and intensity. Further, related limitations include that we were unable to measure overall capacity of these schemes, and participation among ethnic and socioeconomic groups experiencing higher rates of childhood obesity, and by gender. These are important demographic categories which process and outcome evaluations should address.

Notwithstanding these limitations, our database serves to provide a starting point from which to track the extent to which the evidence base is being used in the design and implementation of interventions. Development of the evidence base is more likely to be achieved if funding is linked to a common evaluation framework such as the recently published Standard Evaluation Framework for weight management interventions[22].

## Conclusions

The mapping approach we used is a relatively efficient and effective way of collating information about service activity across a wide range of providers and sectors. Such an approach may be useful in other areas of health or social care, particularly where diverse providers exists and where reliable information needs to be collected rapidly.

There appeared to be great duplication of effort in the design and establishment of similar intervention models, and in many cases a need for more rigorous evaluation.

## Abbreviations

BMI: Body Mass Index; HIV: Human Immunodeficiency Virus; NICE: National Institute for Health and Clinical Excellence; PCT: Primary Care Trust.

## Competing interests

The authors declare that they have no competing interests.

## Authors' contributions

CA carried out the majority of the fieldwork and led the first draft of this paper; LA designed the study, drawing on work she had done on sexual health at the Child Health Research and Policy Unit, City University, London. HR took responsibility for the final report. All authors contributed to the writing of this paper. All authors read and approved the final manuscript.

## Authors' information

At the time the research was carried out, all authors were at the Institute of Education's Social Science Research Unit/EPPI-Centre. At the time of submission CA is at University College London (UCL), LA at Teesside University and HR at UCL Institute of Child Health.

## Pre-publication history

The pre-publication history for this paper can be accessed here:

http://www.biomedcentral.com/1471-2458/10/310/prepub

## Supplementary Material

Additional file 1 **Table S1 - Coding Framework: data extraction guidelines for the mapping exercise**. This file contains the framework used to create a standardised description each scheme which met the inclusion criteria for the mapping exercise. Click here for file

Additional file 2 **Table S2 - Mapping exercise - selected scheme details**. This file displays a selection of the data collected on each of the 51 schemes included in the mapping exercise. Click here for file
