# Supplementary material for: Mapping service activity: the example of childhood obesity schemes in England
Source: BMC Public Health. 2010 Jun 4;10:310. doi: 10.1186/1471-2458-10-310 (PMC2890690; doi:10.1186/1471-2458-10-310)
Supplement: Additional file 1 — Table S1 - Coding Framework: data extraction guidelines for the mapping exercise. This file contains the framework used to create a standardised description each scheme which met the inclusion criteria for the mapping exercise. [file 1471-2458-10-310-S1.DOC]

**Table S2 – Coding Framework: data extraction guidelines for the mapping exercise**

Priority questions are marked with asterisks*

| **Section A: Background** *Covers: basic details about the scheme, its setting up, links with other initiatives and possible roll out.*   | A.1 Name of scheme | | A.1.1 Enter name *(free text)* | | --- | | | --- | --- | --- | | A.2 Telephone number | | A.2.1 Enter telephone number *(free text)* | | --- | | | A.3 Website | | A.3.1 Enter website address *(free text)* | | --- | | | A.4 Address of scheme | | A.4.1 Enter address *(free text)* | | --- | | | A.5 Contact name(s) | | A.5.1 Enter contact name(s) *(free text)* | | --- | | | A.6 Email address(es) | | A.6.1 Enter email address(es) *(free text)* | | --- | | | A.7 When was the scheme set up? *(For schemes in development, enter "2008+")* | | A.7.1 Pre-2002 | | --- | | A.7.2 2002 | | A.7.3 2003 | | A.7.4 2004 | | A.7.5 2005 | | A.7.6 2006 | | A.7.7 2007 | | A.7.8 2008+ | | A.7.9 Unknown | | | A.8 *Is the scheme still running? | | A.8.1 No | | --- | | A.8.2 Yes | | A.8.3 Unknown | | | A.9 *Who are the key partners, including those involved in the scheme's set up? *List, indicating the lead organisation.* | | A.9.1 LEAD ORGANISATION *(please specify)* | | --- | | A.9.2 Primary Care Trust (PCT) | | A.9.3 Local Authority (LA) | | A.9.4 School(s) | | A.9.5 Other | | A.9.6 Voluntary organisation | | A.9.7 Unknown | | |
| --- | --- | --- | --- | --- | --- | --- | --- | --- | --- | --- | --- | --- | --- | --- | --- | --- | --- | --- | --- | --- | --- | --- | --- | --- | --- | --- | --- | --- | --- | --- | --- | --- | --- | --- | --- | --- | --- | --- | --- | --- | --- | --- | --- |
| **Section B: Overall approach** *Covers the objectives and main components of the programme, target age group, who delivers the intervention, the involvement of parents and others, and the programme's evidence base.*   | B.1 *What are the programme's (stated) objectives? | | B.1.1 Please add details *(free text)* | | --- | | B.1.2 Not stated but implicit: weight management or weight loss | | | --- | --- | --- | --- | | B.2 *What age group does the intervention cover? *(Age group of target population)* | | B.2.1 age range unknown, but exclusive to children and/or young people | | --- | | B.2.2 Other details *(please specify)* | | B.2.3 4 | | B.2.4 5 | | B.2.5 6 | | B.2.6 7 | | B.2.7 8 | | B.2.8 9 | | B.2.9 10 | | B.2.10 11 | | B.2.11 12 | | B.2.12 13 | | B.2.13 14 | | B.2.14 15 | | B.2.15 16 | | B.2.16 17 | | B.2.17 18 | | B.2.18 unknown | | | B.3 *Which of the following does the intervention cover? *Any number of components can be identified.* | | B.3.1 Behaviour change techniques | | --- | | B.3.2 Advice/information about healthy eating | | B.3.3 Advice/information about physical activity | | B.3.4 Some form of physical activity | | B.3.5 Healthy food preparation/cooking, and/or tasting/provision | | B.3.6 Other *(please specify)* | | B.3.7 Unknown | | | B.4 Who delivers the intervention? *(select all that apply)* | | B.4.1 Unknown | | --- | | B.4.2 Community Worker | | B.4.3 Counsellor | | B.4.4 Health professional: dietician/nutritionist | | B.4.5 Health professional: School nurse | | B.4.6 Health professional: other *(please specify)* | | B.4.7 Health promotion/education practitioner | | B.4.8 Lay therapist | | B.4.9 Parent | | B.4.10 Peer *(please specify)* | | B.4.11 Psychologist | | B.4.12 Researcher | | B.4.13 Residential worker | | B.4.14 Social worker | | B.4.15 Sports/exercise worker | | B.4.16 Teacher/lecturer | | B.4.17 Other *(please specify)* | | | B.5 Are parents/carers/other family members involved in the intervention? | | B.5.1 Yes | | --- | | B.5.2 No | | B.5.3 Unknown | | | B.6 If yes, how are they involved? *(select all that apply)* | | B.6.1 n/a *(not involved or unknown)* | | --- | | B.6.2 Unknown | | B.6.3 Attend scheme with child | | B.6.4 Other *(please specify)* | | B.6.5 Cook with the child | | B.6.6 Support child at home with eating and/or exercise | | B.6.7 Go food shopping with child | | | B.7 Was information and/or advice used in the setting up of the intervention? | | B.7.1 Yes | | --- | | B.7.2 No | | B.7.3 Unknown | | | B.8 If yes, what was this advice/information? *(if 'yes' to previous question, select all that apply)* | | B.8.1 Analysis of local needs | | --- | | B.8.2 Specific named theory/model of behaviour change referred to in setting up/running the intervention | | B.8.3 Experience of other interventions | | B.8.4 Expert advice | | B.8.5 NICE 2006 Guidance | | B.8.6 Other research evidence | | B.8.7 Other *(please specify)* | | B.8.8 n/a | | |
| **Section C: Running the intervention** *Covers the duration of the intervention and the settings where it is delivered.*   | C.1 *On average, and based on the most recent data, how long does the core period of the intervention last? *(also specify if any additional 'follow-up' sessions take place)* | | C.1.1 Core period duration unknown | | --- | | C.1.2 Less than one week | | C.1.3 One week | | C.1.4 Two to six weeks | | C.1.5 Seven to nine weeks | | C.1.6 Ten to twelve weeks | | C.1.7 More than twelve weeks | | C.1.8 Unknown if there is any follow-up | | C.1.9 No additional follow-up | | C.1.10 Additional one-off follow-up | | C.1.11 Other follow-up *(please specify)* | | | --- | --- | --- | --- | --- | --- | --- | --- | --- | --- | --- | --- | --- | | C.2 What kind of venue is the intervention delivered in? *(select as many settings as apply)* | | C.2.1 Unknown | | --- | | C.2.2 Community *(please specify)* | | C.2.3 Correctional institution | | C.2.4 Day care centre | | C.2.5 Educational institution – unspecified | | C.2.6 Educational institution - pre-school | | C.2.7 Educational institution - primary education | | C.2.8 Educational institution - secondary education | | C.2.9 Educational institution - FE/college | | C.2.10 Family centre | | C.2.11 Health care unit - unspecified | | C.2.12 Health care unit - primary care | | C.2.13 Health care unit - hospital | | C.2.14 Health care unit - specialist clinic | | C.2.15 Home | | C.2.16 Hospice | | C.2.17 Leisure Centre | | C.2.18 Outreach | | C.2.19 Residential care | | C.2.20 Residential outing *(e.g. summer camp)* | | C.2.21 Workplace *(please specify)* | | C.2.22 Other *(please specify)* | | |

| **Section D: Recruitment/referral to scheme** *Covers the process by which those using the scheme come to use it, local/national knowledge of the scheme, the sex of users, completion rates, the number of people who are able to use the service.*   | D.1 *What is the main referral route? | | D.1.1 Unknown | | --- | | D.1.2 Referral by GP | | D.1.3 Referral by parent | | D.1.4 Referral by school nurse | | D.1.5 Referral by social worker | | D.1.6 Referral by teacher | | D.1.7 Self referral | | D.1.8 Other *(please specify)* | | | --- | --- | --- | --- | --- | --- | --- | --- | --- | --- | | D.2 *What are the weight-related admission criteria? *(select all that apply)* | | D.2.1 Not known - but overweight and/or obese | | --- | | D.2.2 Waist/hip ratio *(please add details)* | | D.2.3 Waist circumference *(please add details)* | | D.2.4 Other *(please specify)* | | D.2.5 BMI percentile for age and sex above 99 | | D.2.6 BMI percentile for age and sex above 98 | | D.2.7 BMI percentile for age and sex above 97 | | D.2.8 BMI percentile for age and sex above 95 | | D.2.9 BMI percentile for age and sex above 91 | | D.2.10 'obese' | | D.2.11 'overweight or obese' | | | D.3 Is the intervention restricted to specific groups? *(If yes, which groups?)* | | D.3.1 Unknown | | --- | | D.3.2 No | | D.3.3 Yes - BME groups | | D.3.4 Yes - low-income children | | D.3.5 Yes - children with learning disability | | D.3.6 Yes - children with SEN | | D.3.7 Yes - parents must accompany child to session | | D.3.8 Yes - parents must be involved at home/other setting | | D.3.9 Yes - girls only | | D.3.10 Yes - boys only | | D.3.11 Yes – other *(please specify)* | | | D.4 *How many children can the programme cover per year? *(Programme's capacity, based on most recent figures)* | | D.4.1 Enter number *(free text)* | | --- | | D.4.2 Unknown | | | D.5 How many children participate per year? *(Based on most recent figures)* | | D.5.1 Enter number *(free text)* | | --- | | D.5.2 Unknown | | |
| --- | --- | --- | --- | --- | --- | --- | --- | --- | --- | --- | --- | --- | --- | --- | --- | --- | --- | --- | --- | --- | --- | --- | --- | --- | --- | --- | --- | --- | --- | --- | --- | --- | --- | --- | --- | --- | --- | --- | --- | --- | --- | --- | --- | --- |
| **Section E: Costs and funding**   | E.1 *How is the intervention funded? *(including contributions in kind)* | | E.1.1 Unknown | | --- | | E.1.2 Funding from charities/voluntary organisations | | E.1.3 Funding from DH | | E.1.4 Other funding *(please specify)* | | E.1.5 PCT funding | | E.1.6 PCT contributions in kind | | E.1.7 LA funding | | E.1.8 LA contributions in kind | | E.1.9 School contributions in kind, e.g. provision of venue | | E.1.10 Other contributions in kind | | E.1.11 Payment from family | | | --- | --- | --- | --- | --- | --- | --- | --- | --- | --- | --- | --- | --- | |
| **Section F: Monitoring and evaluation** *Covers data collection, follow-up, effectiveness, evidence used to set up the scheme, changes to the scheme and challenges to running of the scheme.*   | F.1 *Has monitoring/evaluation data been collected about the intervention? | | F.1.1 Yes | | --- | | F.1.2 No | | F.1.3 Unknown | | F.1.4 Yes - provided | | F.1.5 Yes - but not provided | | F.1.6 In process | | | --- | --- | --- | --- | --- | --- | --- | --- | | F.2 If 'yes', where can this be located? *(please specify)* | | F.2.1 Enter details *(free text)* | | --- | | | F.3 *What kind of outcomes are measured in the monitoring/evaluation document? *(select all that apply)* | | F.3.1 Changes in BMI | | --- | | F.3.2 Changes in waist measurement | | F.3.3 Changes in weight | | F.3.4 Knowledge re: healthy eating | | F.3.5 Other *(please specify)* | | F.3.6 Unknown | | | F.4 In the evaluation/monitoring document, is there any discussion of the main levers and barriers to intervention implementation and effectiveness? | | F.4.1 Yes | | --- | | F.4.2 No | | F.4.3 Unknown | | | F.5 If 'yes', what are the main levers and barriers identified? *(please specify)* | | F.5.1 Main levers *(free text)* | | --- | | F.5.2 Main barriers *(free text)* | | |
|  |
